# Expression of Autotaxin–Lysophosphatidate Signaling-Related Proteins in Breast Cancer with Adipose Stroma

**DOI:** 10.3390/ijms20092102

**Published:** 2019-04-29

**Authors:** Yoon Jin Cha, Ja Seung Koo

**Affiliations:** Department of Pathology, Severance Hospital, Yonsei University College of Medicine, 50 Yonsei-ro, Seodaemun-gu, Seoul 03722, Korea; yooncha@yuhs.ac

**Keywords:** adipocyte, autotaxin, breast cancer, lysophosphatidate, stroma

## Abstract

This research aimed to evaluate the expression and clinical implication of autotaxin (ATX)-lysophosphatidate (LPA) signaling-related proteins in breast cancer with adipose stroma. To this end, a tissue microarray (TMA) was constructed from 137 breast cancer tissues with adipose stroma and 329 breast cancer tissues with non-adipose stroma (inflammatory stroma: *n* = 81, 24.6%; fibrous stroma: *n* = 246, 75.4%). Immunohistochemical staining for ATX-LPA signaling-related proteins (ATX, LPA1, LPA2, and LPA3) was performed on the TMA. The results showed that LPA2 in tumor cells and LPA3 in stromal cells were highly expressed in breast cancer with adipose stroma and breast cancer with adipose and inflammatory stroma, respectively. Stromal LPA1 positivity (*p* = 0.017) and stromal LPA3 positivity (*p* = 0.004) were higher in breast cancer with adipose stroma containing CD68-positive crown-like structures (CLS). Stromal ATX positivity (*p* = 0.010) and stromal LPA3 positivity (*p* = 0.009) were higher in breast cancer with adipose tissue containing CD163-positive CLS. In breast cancer with adipose stroma, the number of CD163-positive macrophages was greater with stromal ATX positivity (*p* = 0.003), and the number of CD68-positive and CD163-positive macrophages were greater in cases with stromal LPA3 positivity. In conclusion, ATX-LPA signaling-related proteins are highly expressed in breast cancer with adipose stroma, with associated macrophage infiltration.

## 1. Introduction

Autotaxin (ATX) is a glycoprotein encoded by the ENPP2 gene located on chromosome 8 [1]. Identical to lysophospholipase D, ATX plays a role in the synthesis of the bioactive lipid mediator lysophosphatidate (LPA) from lysophosphatidylcholine (LPC). LPA binds to LPA receptors; activates phospholipase C and the MAPK, PI3K, and PhoA pathways; and is involved in various cellular processes [2,3]. LPA receptors are G-protein coupled receptors, and at least six LPA receptors (LPA1-LPA6) have been identified. LPA1-LPA3 belong to EDG family (LPA1-EDG2, LPA2-EDG4, LPA3-EDG7), and LPA4-LPA6 have a similar structure to the P2Y nucleotide receptor [4]. The ATX-LPA signaling axis is closely related to tumor biology, including tumor formation, progression, and metastasis [5,6]. ATX is generated from platelets, endothelial cells, fibroblasts, and adipocytes [7,8,9,10], and specifically, ATX from adipocytes has an impact on plasma LPA level [11]. Thus, adipocytes could be an important origin of ATX in tumors. Breast cancer is a human cancer that has adipocyte-rich stroma. Adipose tissue comprises 56% of non-lactating breast [12] tissue, and 35% of lactating breast tissue [13]. ATX-LPA signaling has been reported to be involved in angiogenesis, tumor cell invasion, and migration in breast cancer [14].

Therefore, breast cancer with adipose stroma may differ in the expression of ATX-LPA signaling-related proteins, and this possibility has not been investigated thoroughly. In this study, we evaluated the expression and clinical implications of ATX-LPA signaling-related proteins in breast cancer with adipose stroma.

## 2. Materials and Methods

### 2.1. Patient Selection and Histologic Evaluation

A total of 137 breast cancer with more than 50% adipose stroma were collected from data files of the Department of Pathology of Severance Hospital, Seoul, Korea. For comparison, 329 cases of breast cancer with non-adipose stroma were included in the study. Only patients with a diagnosis of invasive ductal carcinoma were included. All slides were reviewed after collection and pathologic diagnoses were confirmed by two pathologists (Ja Seung Koo and Yoon Jin Cha). The histological grade was assessed using the Nottingham grading system [15].

For stroma, the boundaries of the invasive tumor were identified and stroma inside them was evaluated. If more than 50% of stroma was adipose tissue, then it considered as breast cancer with adipose stroma. Cases with less than 50% of adipose stroma were divided into two subgroups according to the type of stroma: inflammatory stroma and fibrous stroma. Cases having more than 50% of stromal tumor infiltrating lymphocytes (TILs) were defined as breast cancer with inflammatory stroma. The remainder were separately defined as breast cancer with fibrous stroma. Evaluation of stromal TIL were according to the guideline of TIL working group [16].

### 2.2. Tissue Microarray (TMA)

A representative area showing tumor and tumor stroma was selected on a hematoxylin and eosin (H&E)-stained slide, and the corresponding spot was marked on the surface of a paraffin block. Using a biopsy needle, the selected area was punched out, and a 3-mm tissue core was transferred to a 6 × 5 recipient block. Two cores of invasive tumor tissue were extracted to minimize extraction bias. Each tissue core was assigned a unique TMA location number that was linked to a database containing other clinicopathologic data. Cases showed different tumor stromal nature after multiple sections of TMA block were excluded.

### 2.3. Immunohistochemistry

The antibodies used for immunohistochemistry (IHC) in this study are shown in Table 1. Tissue sections 3-μm in thickness from formalin-fixed, paraffin-embedded (FFPE) tissue was deparaffinized and rehydrated using a xylene and alcohol solution. IHC was performed using a Ventana Discovery XT automated stainer (Ventana Medical System, Tucson, AZ, USA). Cell Conditioning 1 buffer (CC1; citrate buffer pH 6.0, Ventana Medical System) was used for antigen retrieval. Appropriate positive and negative controls were included. The positive controls are used as manufacturer’s suggestion (ATX; human tonsil tissue, LPA1; human colon tissue, LPA2; Breast cancer tissue, LPA3; Human prostate carcinoma).

### 2.4. Interpretation of Immunohistochemical Staining

All immunohistochemical markers were accessed by light microscopy. A cut-off value of at least 1% positively stained nuclei was used to define estrogen receptor (ER) and progesterone receptor (PR) positivity [17]. Human epidermal growth factor receptor (HER)2 staining was analyzed according to the American Society of Clinical Oncology (ASCO)/College of American Pathologists (CAP) guidelines using the following categories: 0 = no immunostaining; 1+ = weak incomplete membranous staining, less than 10% of tumor cells; 2+ = complete membranous staining, either uniform or weak in at least 10% of tumor cells; and 3+ = uniform intense membranous staining in at least 30% of tumor cells [18]. HER2 immunostaining was considered positive when strong (3+) membranous staining was observed, whereas cases with 0 to 1+ were regarded as negative. Cases showing 2+ HER2 expression were evaluated for HER2 amplification using fluorescent in situ hybridization (FISH). Ki-67 labeling index (LI) was defined as the percentage of positive nuclear stained tumor cells.

IHC for ATX-LPA signaling-related proteins was evaluated in a semi-quantitative manner [19]. Tumor and stromal cell staining were assessed as follows: 0 = negative or weak immunostaining in <1% of the tumor/stroma, 1 = focal expression in 1–10% of tumor/stroma, 2 = positive in 11%–50% of tumor/stroma, and 3 = positive in 51%–100% of tumor/stroma. The whole tumor area was evaluated, and a score of 0, 1 was assigned as negative and scores of 2 and higher were assigned as positive.

### 2.5. Western Blotting

Deparaffinization and protein extraction of FFPE tissue was carried out using Qproteome FFPE Tissue Kit (Qiagen, Hilden, Germany; #1042481) following manufacturer’s instructions. Extracted proteins were determined by Bradford assay (BIO-RAD, CA, USA; #5000205). Quantified proteins were mixed with 5 × SDS-PAGE loading buffer (Biosesang, Seongnam, Korea; S2002) in concentration of 2 μg/μL and boiled at 95 °C for 5 min. Equal quantities of protein were separated to SDS-PAGE gel and transferred to nitrocellulose membranes (BIO-RAD; #1704158). Membranes were blocked by incubation in 5% skim milk in Tris-buffered saline (TBS) with 0.1% Tween-20 and probed with antibody against ATX (1:1000; Abcam, Cambridge, UK; ab140915), LPA1(1:2000; Abcam; ab166903), and LPA2(1:1000; Abcam; ab38322) diluted in 1% BSA in TBS, 0.1% Tween 20, and 0.02% NaN_3_. The membranes were washed and then incubated with secondary antibodies (HRP conjugated anti-mouse IgG, or anti-rabbit IgG) (1:20,000; Santa Cruz, TX, USA) for 1 h at room temperature. The bands were visualized using WesternBright ECL (Advansata, CA, USA; K-12045-D50) after washing the membrane and exposed to X-ray film.

### 2.6. Tumor Phenotype Classification

We classified the breast cancer phenotypes according to the immunohistochemistry results for ER, PR, HER2, and Ki-67, and the silver in situ hybridization results for HER2, as follows [20]:

* luminal A type: ER- or PR-positive, or both, HER2-negative

* luminal B type: (HER2-negative) ER- or PR-positive, or both, HER2-negative, and Ki-67 LI ≥14%;

(HER2-positive) ER- or PR-positive, or both, and HER2overexpressed or amplified, or both

* HER2 type: HER2-overexpressed or amplified, or both, regardless of ER and/or PR status

* Triple Negative Breast Cancer (TNBC) type: ER-, PR-, and HER2-negative

## 3. Statistical Analysis

Data were analyzed using SPSS for Windows, Version 23.0 (SPSS Inc., Chicago, IL, USA). For determination of statistical significance, the Student’s *t* and Person chi-square tests were used for continuous and categorical variables, respectively. In the case of analyzing data with multiple comparisons, a corrected *p*-value with the application of the Bonferroni multiple comparison procedure was used. Correlation analysis was evaluated using the Pearson correlation coefficient (*r*). Statistical significance was set to *p* < 0.05. Kaplan–Meier survival curves and log-rank statistics were employed to evaluate time to tumor recurrence and overall survival. Multivariate regression analysis was performed using the Cox proportional hazards model.

## 4. Results

### 4.1. Characteristics of Patients According to Breast Cancer Stroma Type

There was a total of 137 (29.4%) breast cancer tissues containing adipose stroma and 329 (70.6%) with non-adipose stroma, with non-adipose stroma subdivided into inflammatory stroma (*n* = 81, 24.6%), and fibrous stroma (*n* = 248, 53.2%). Histologic grade, ER status, PR status, HER2 status, and molecular subtype were significantly different between groups (*p* < 0.001). Breast cancer with inflammatory stroma had a higher histologic grade, and frequently showed ER negativity, PR negativity, HER2 positivity, and non-luminal A type (Table 2).

### 4.2. Expression of ATX-LPA Signaling-Related Proteins according to Breast Cancer Stroma Type

**Figure 1:**
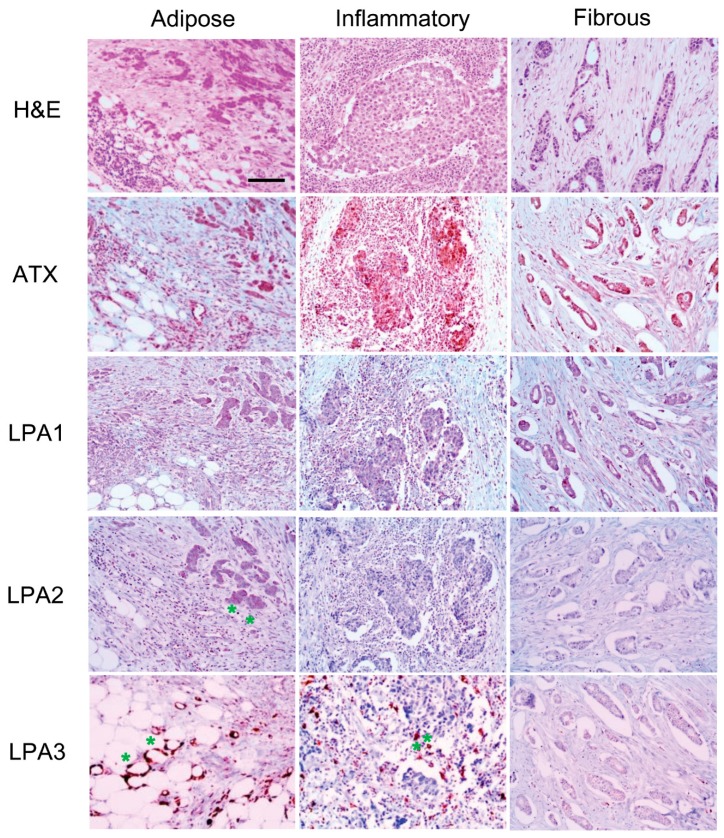
Expression of ATX-LPA signaling-related proteins according to breast cancer stroma type in immunohistochemical stain. High expression of LPA2 in cancer cells is observed in the breast cancer with adipose stroma (*), and LPA3 is highly expressed in the stromal cell compartment of breast cancer with adipose stroma and breast cancer with inflammatory stroma (*). LPA3 positive stromal cells are immune cells like macrophages. H&E, hematoxylin and eosin. Scale bar represents 200 μm.

**Figure 2:**
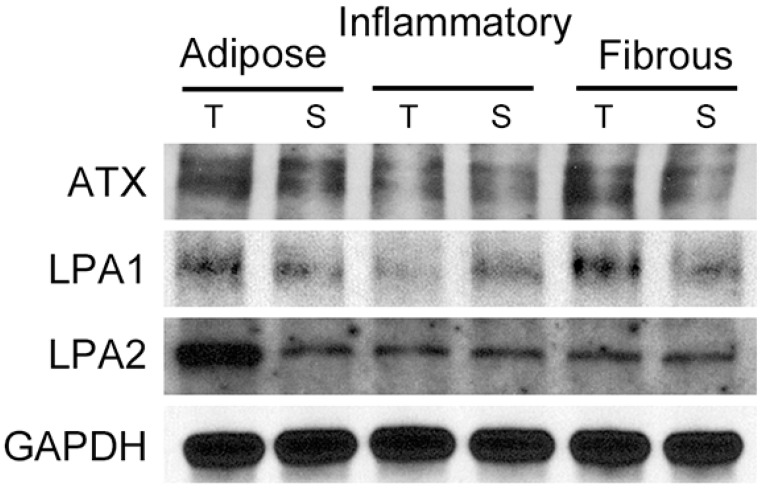
Expression of ATX-LPA signaling-related proteins according to breast cancer stroma type in Western blot. ATX and LPA1 expressions had no significant differences among stroma types or cancers around them. However, level of LPA2 of tumor around adipose stroma was much higher than the other tumors surrounded by inflammatory or fibrous stroma. T, tumor; S, stroma.

ATX, LPA1, and LPA3 were expressed in tumor cells as well as stromal cells, whereas LPA2 was only expressed in tumor cells. LPA2 in tumor cells and LPA3 in stromal cells were differentially expressed according to the type of breast cancer stroma. LPA2 was highly expressed in breast cancer with adipose stroma. Stromal LPA3 was highly expressed in breast cancer with adipose stroma and inflammatory stroma. Immune cells, such as macrophages, were stromal cells that were positive for LPA3 (Table 3, Supplementary Figure S1 and Figure 1). In Western blot analysis, the ATX and LPA1 expressions had no significant differences among stroma types nor cancers around them. However, level of LPA2 of tumor around adipose stroma was much higher than the other tumors surrounded by inflammatory or fibrous stroma (Figure 2).

### 4.3. Correlation of the Expression of ATX-LPA Signaling-Related Proteins

Positive correlations were found in ATX-stromal ATX (*r* = 0.348, *p* < 0.001), ATX-LPA1 (*r* = 0.367, *p* < 0.001), ATX-stromal LPA1 (*r* = 0.125, *p* = 0.007), ATX-LPA2 (*r* = 0.159, *p* = 0.001), stromal ATX-LPA1 (*r* = 0.141, *p* = 0.002), stromal ATX-stromal LPA1 (*r* = 0.352, *p* < 0.001), stromal ATX-stromal LPA3 (*r* = 0.121, *p* = 0.009), LPA1-stromal LPA1(*r* = 0.273, *p* < 0.001), LPA1-LPA2 (*r* = 0.221, *p* < 0.001), LPA1-stromal LPA3 (*r* = 0.216, *p* < 0.001), and stromal LPA1-stromal LPA3 (*r* = 0.291, *p* < 0.001) (Table 4).

### 4.4. Correlation between the Expression of ATX-LPA Signaling-Related Proteins and Macrophages in Adipose Stroma

**Figure 3:**
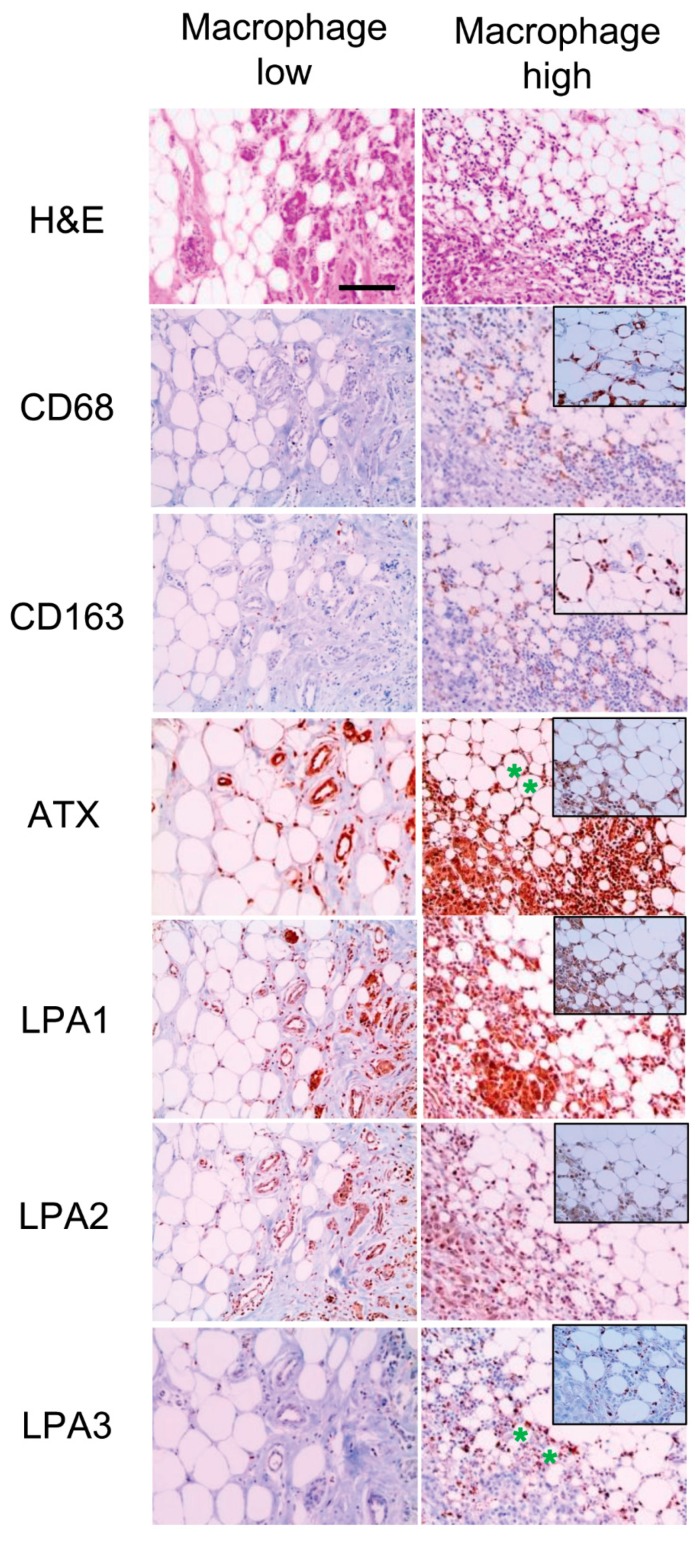
Correlation between the expression of ATX-LPA signaling-related proteins and macrophages in adipose stroma. Increased ATX expression in stromal cells is associated with increased number of CD163-positive macrophages (*). Increased LPA3 expression in stromal cells is associated with high numbers of both CD68- and CD163-positive macrophages (*). Note CD68 and CD163-positive CLS (inlet). Scale bar represents 200 μm.

We have previously reported infiltrating macrophages in adipose stroma of breast cancer recognized by CD68 and CD163 IHC [21], and the results of the previous study were used to analyze this relationship with the expression of ATX-LPA signaling-related proteins. CD68-positive crown-like structure (CLS) was correlated with a higher rate of stromal LPA1 positivity (*p* = 0.017) and stromal LPA3 positivity (*p* = 0.004). CD163-positive CLS was correlated with higher stromal ATX positivity (*p* = 0.010) and stromal LPA3 positivity (*p* = 0.009) (Table 5 and Figure 2). Stromal ATX positivity was correlated with the number of CD163-positive macrophages infiltrating the adipose stroma (*p* = 0.003). Stromal LPA3 positivity was correlated with number of both CD68- and CD163-positive macrophages (*p* < 0.001, Table 6 and Figure 3).

### 4.5. ATX-LPA Signaling-Related Proteins and Clinicopathologic Parameters

**Figure 4:**
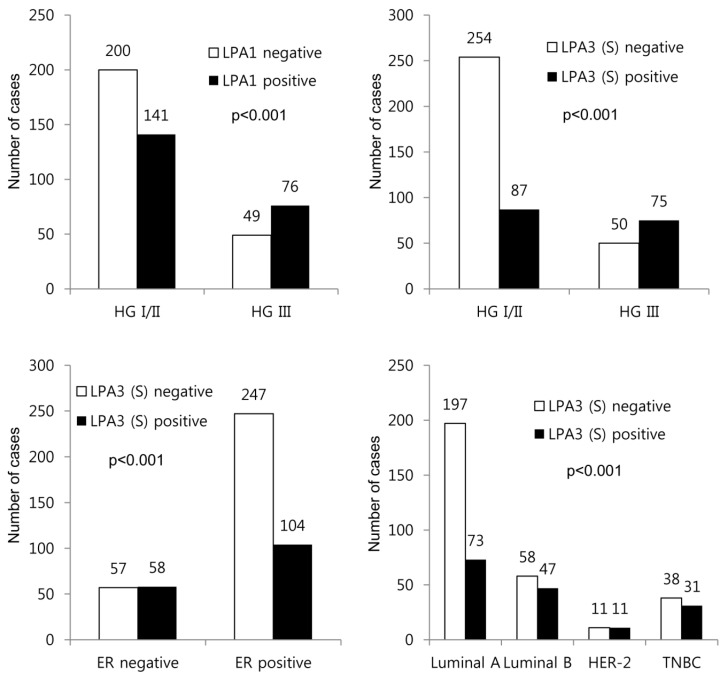
Correlation between clinicopathologic parameters and the expression of ATX-LPA signaling-related proteins. LPA1 positivity is associated with higher histologic grade (*p* < 0.001), and stromal LPA3 positivity is associated with higher histologic grade (*p* < 0.001), ER negativity (*p* < 0.001), and non-luminal A type (*p* = 0.001). HG, histologic grade.

LPA1 positivity was associated with higher histologic grade (*p* < 0.001) and stromal LPA3 positivity was associated with higher histologic grade (*p* < 0.001), ER negativity (*p* < 0.001), and non-luminal A type (*p* = 0.001) (Figure 4 and Supplementary Table S1). In further subgroup analysis for breast cancer with adipose stroma and non-adipose stroma, stromal LPA3 positivity showed significant association with higher histologic grade in adipose stroma group (*p* < 0.001), and higher histologic grade (*p* < 0.001), ER negativity (*p* < 0.001), and non-luminal A type (*p* = 0.001) in non-adipose stroma group (Supplementary Figure S2).

### 4.6. Impact of the Expression of ATX-LPA Signaling-Related Proteins on Patient Prognosis

**Figure 5:**
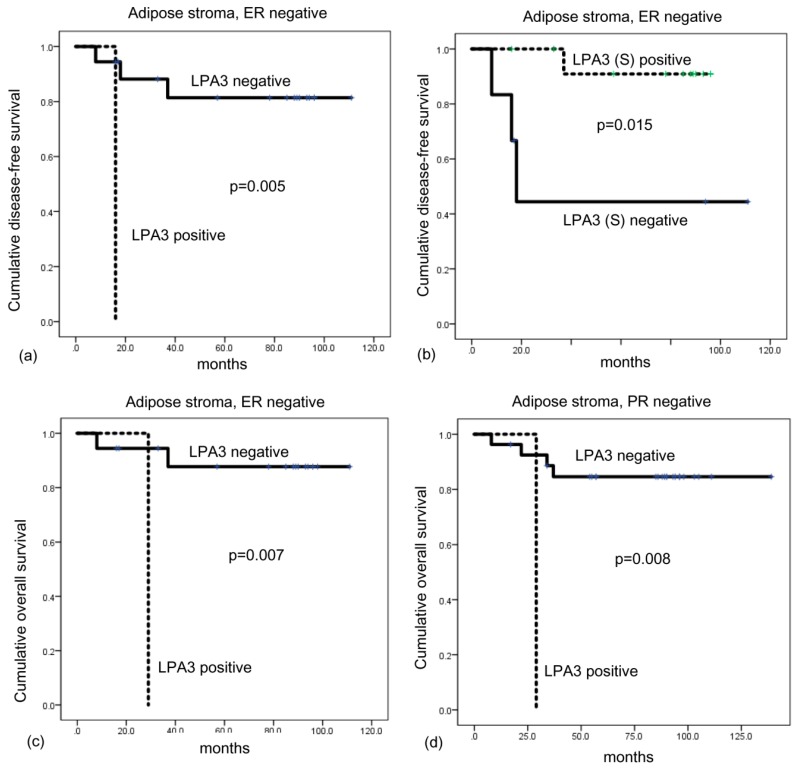
Disease-free survival and overall survival according to LPA3 expression in breast cancer with adipose stroma. In breast cancer with adipose stroma and ER-negative breast cancer, LPA3 positivity (**a**) and stromal LPA3 negativity (**b**) are associated with shorter disease-free survival, and LPA3 positivity is associated with shorter overall survival (OS) (**c**). LPA3 positivity is associated with shorter OS in breast cancer with adipose stroma and PR-negative breast cancer (**d**). Green, censored LPA3(S) positive patients; blue, censored LPA3 negative patients

On univariate analysis, expression of ATX-LPA signaling-related proteins and patient prognosis showed no significant association (Table 7). However, univariate analysis within subgroups showed that shorter disease-free survival was associated with LPA positivity (*p* = 0.005) and stromal LPA3 negativity (*p* = 0.015) in breast cancer with adipose stoma and ER negative cases, and LPA3 positivity (*p* = 0.007) was associated with shorter overall survival (Figure 3). LPA3 positivity (*p* = 0.008) was associated with shorter overall survival in breast cancer with adipose stroma and PR negative cases (Figure 5).

## 5. Discussion

We investigated the expression of ATX-LPA signaling-related proteins in breast cancer with adipose stroma. Compared with breast cancer with non-adipose stroma, breast cancer with adipose stroma had higher LPA2 expression of tumor cells. Previous studies have shown higher expression of ATX and LPA1, 2, and 3 in breast cancer compared with normal breast tissue [22,23,24,25]. In human breast cancer, tumor cells show higher expression of LPA2 than LPA1 and LPA3 in postmenopausal breast cancer tissue [26]. However, previous studies have lacked information regarding ATX-LPA signaling-related protein expression according to the stromal type. Higher LPA2 expression in tumor cells of breast cancer with adipose stroma may be affected by interactions between cancer cells and cancer-associated adipocytes (CAAs). Various cross-talk and interactions are present between breast cancer cells, stromal cells, and adipocytes [27]. Between adipocytes, one of the stromal components of breast cancer, these interactions affect tumor biology [28]. Increased secretion of various adipokines by CAAs in close proximity to tumor cells has an effect on tumor cells. Conversely, cytokines and growth factors secreted from tumor cells also influence adipocytes in the vicinity, which is a reciprocal interaction between breast cancer cells and adjacent adipocytes [29]. Hence, CAAs may play a role in the high expression of LPA2 in breast cancer cells, which requires further study. A previous study reported that LPA2 of breast cancer cells regulates HIF-1α expression and is involved in cell proliferation, migration, and invasion [30]. LPA2 expression was associated with nodal metastasis and higher stage [30]. Therefore, higher expression of LPA2 in tumor cells of breast cancer with adipose stroma may have an effect on breast cancer biology.

In the present study, stromal LPA3 was highly expressed in breast cancer with adipose stroma and breast cancer with inflammatory stroma, and was particularly positive in the immune cells, such as macrophages. Previous studies have also recognized that expression of ATX-LPA signaling-related proteins was found in stromal cells, mostly fibroblasts [22,24,25,31]. We also identified protein expression in fibroblasts, as well as in immune cells. The ATX-LPA receptor axis affects breast cancer-related inflammation; in MMTV-LPR transgenic mice, expression of ATX, LPA1, LPA2, and LPA3 for mammary cancer induction was associated with inflammation in mammary tissue, even before tumor development [32]. ATX secreted from tumor and stromal cells induces LPA, which enhances NF-κB expression via the PKC or AKT pathway, induces ATF-2 via the Rho-CDC42-p38 MAPK pathway, and activates STAT3 and STAT5, which induces the synthesis of inflammatory cytokines, chemokines, and COX-2 [32,33,34,35]. Thus, the ATX-LPA receptor axis generates inflammatory factors and facilitates an inflammatory tumor microenvironment.

We observed LPA3 expression in immune cells in breast cancer with adipose stroma or inflammatory stroma. Stromal LPA3 positivity was associated with CD68- and CD163-positive CLS, and correlated with CD68- and CD163-positive macrophages infiltrating in adipose tissue. As macrophages were frequently positive for LPA3, LPA3 may be associated with tumor-associated macrophages. Among the tumor microenvironment of breast cancer, tumor-associated macrophages (TAMs) are a predominant component of tumor mass, and a subset of breast cancer comprises more than 50% TAMs [36]. Further study on LPA3 positive TAMs is required, as TAMs in breast cancer are involved in various processes, such as tumor progression, metastasis, immune alteration, drug resistance, and cancer stem cells [37].

The present study reports an association between LPA3 positivity and poor prognosis in ER- and PR-negative breast cancer with adipose stroma. In a previous study, LPA3 expression in breast cancer was associated with high tumor grade, HER2 positivity, and lymph node metastasis [24]. Moreover, LPA3 transgenic mice showed high rate of metastasis [32], suggesting that LPA is an indicator of poor prognosis in breast cancer, which needs to be validated in a future study.

The ATX-LPA axis can be a therapeutic target of breast cancer. Since tumor and stromal cells in breast cancer express ATX-LPA signaling-related proteins, inhibition of the ATX-LPA axis could have a dual effect. Ki16245, a non-lipid competitive inhibitor of LPA and LPA3, reduces bone metastasis of breast cancer in a mouse model [10]. Debio0719, an R-stereoisomer of Ki16425, reduced lung and bone metastasis [38] and liver and lung metastasis [39] in a mouse model. BrP-LPA, a dual ATX and pan-LPAR inhibitor, inhibited migration and invasion of breast cancer cell lines and suppressed primary tumor and angiogenesis in a mouse xenograft study [40]. ONO-843050, an ATX inhibitor, reduced tumor growth of breast cancer in a mouse model, and had a subsequent decrease in lung metastasis by up to 60% [41]. Therefore, further study on the blockade of the ATX-LPA axis in breast cancer with adipose stroma and inflammatory stroma is required.

In conclusion, LPA2 and stromal LPA3 are highly expressed in breast cancer with adipose stroma. Among them, stromal LPA3 expression of breast cancer with adipose stroma is associated with macrophage infiltration.

### 5.1. Compliance with Ethical Standards

#### Ethical Standards

This study was approved by the Institutional Review Board (IRB) of Severance Hospital with wavier of informed consents (IRB No. 4-2016-0832; approval date, 14 September 2016). All procedures performed in studies involving human participants were in accordance with the ethical standards of the institutional and/or national research committee and with the 1964 Helsinki declaration and its later amendments or comparable ethical standards.

## Figures and Tables

**Table 1:** Source, clone, and dilution of antibodies.

Antibody	Company	Clone	Dilution
*ATX-LPA pathway*-*related proteins*			
ATX	Abcam, Cambridge, UK	Polyclonal	1:1000
LPA1	Abcam, Cambridge, UK	EPR9710	1:100
LPA2	Abcam, Cambridge, UK	Polyclonal	1:100
LPA3	Abcam, Cambridge, UK	Polyclonal	1:250
*Molecular subtype*-*related proteins*			
Estrogen receptor (ER)	Thermo Scientific, San Diego, CA, USA	SP1	1:100
Progesterone receptor (PR)	DAKO, Glostrup, Denmark	PgR	1:50
Human epidermal growth factor receptor-2 (HER2)	DAKO, Glostrup, Denmark	Polyclonal	1:1500
Ki-67	Abcam, Cambridge, UK	SP6	1:100

ATX, autotaxin; LPA, lysophosphatidate.

**Table 2:** Clinicopathologic characteristics of patients according to breast cancer stroma type.

Parameter	Total (*n* = 466) (%)	Adipose Stroma (*n* = 137) (%)	Non-Adipose Stroma (*n* = 329) (%)		*p*-Value *	*p*-Value
			Inflammatory (*n* = 81) (%)	Fibrous (*n* = 248) (%)		
**Age (years)**					0.896	0.612
≤50	287 (61.6)	85 (62.0)	46 (56.8)	156 (62.6)		
>50	179 (38.4)	52 (38.0)	35 (43.2)	92 (37.1)		
Histologic grade					**0.045**	**<0.001**
I/II	341 (73.2)	109 (79.6)	43 (53.1)	189 (76.2)		
III	125 (26.8)	28 (20.4)	38 (46.9)	59 (23.8)		
Tumor stage					0.910	0.948
T1	257 (55.2)	75 (54.7)	46 (56.8)	136 (54.8)		
T2/T3	209 (44.8)	62 (45.3)	35 (43.2)	112 (45.2)		
Nodal metastasis					0.936	0.537
Absent	281 (60.3)	83 (60.6)	53 (65.4)	145 (58.5)		
Present	185 (39.7)	54 (39.4)	28 (34.6)	103 (41.5)		
ER					**<0.001**	**<0.001**
Negative	115 (24.7)	19 (13.9)	39 (48.1)	57 (23.0)		
Positive	351 (75.3)	118 (86.1)	42 (51.9)	191 (77.0)		
PR					**0.002**	**<0.001**
Negative	141 (30.5)	28 (20.4)	39 (48.1)	75 (30.2)		
Positive	324 (65.4)	109 (79.6)	42 (51.9)	173 (69.8)		
HER2 status					0.157	**<0.001**
Negative	387 (83.0)	119 (86.9)	55 (67.9)	213 (85.9)		
Positive	79 (17.0)	18 (13.1)	26 (32.1)	35 (14.1)		
Molecular subtype					**<0.001**	**<0.001**
Luminal A	270 (57.9)	107 (78.1)	23 (28.4)	140 (56.5)		
Luminal B	105 (22.5)	14 (10.2)	28 (34.6)	63 (25.4)		
HER2	22 (4.7)	2 (1.5)	9 (11.1)	11 (4.4)		
Triple negative breast cancer (TNBC)	69 (14.8)	14 (10.2)	21 (25.9)	34 (13.7)		

* Comparison of adipose stroma and non-adipose stroma; Bold, *p*-value < 0.05.

**Table 3:** Expression of ATX-LPA signaling-related proteins according to breast cancer stroma type.

Parameter	Total (*n* = 466) (%)	Adipose Stroma (*n* = 137) (%)	Non-Adipose Stroma (*n* = 329) (%)		*p*-Value *	*p*-Value
			Inflammatory (*n* = 81) (%)	Fibrous (*n* = 248) (%)		
**ATX**					0.086	0.062
Negative	243 (52.1)	63 (46.0)	38 (46.9)	142 (57.3)		
Positive	223 (47.9)	74 (54.0)	43 (53.1)	106 (42.7)		
ATX (S)					0.357	0.511
Negative	390 (83.7)	118 (86.1)	69 (85.2)	203 (81.9)		
Positive	76 (16.3)	19 (13.9)	12 (14.8)	45 (18.1)		
LPA1					0.514	0.363
Negative	249 (53.4)	70 (51.1)	49 (60.5)	130 (52.4)		
Positive	217 (46.6)	67 (48.9)	32 (39.5)	118 (47.6)		
LPA1 (S)					0.854	0.951
Negative	382 (82.0)	113 (82.5)	67 (82.7)	202 (81.5)		
Positive	84 (18.0)	24 (17.5)	14 (17.3)	46 (18.5)		
LPA2					**<0.001**	**<0.001**
Negative	409 (87.8)	85 (62.0)	80 (98.8)	244 (98.4)		
Positive	57 (12.2)	52 (38.0)	1 (1.2)	4 (1.6)		
LPA 3					0.451	0.121
Negative	436 (93.6)	130 (94.9)	79 (97.5)	227 (91.5)		
Positive	30 (6.4)	7 (5.1)	2 (2.5)	21 (8.5)		
LPA 3 (S)					**0.027**	**<0.001**
Negative	304 (65.2)	79 (57.7)	34 (42.0)	191 (77.0)		
Positive	162 (34.8)	58 (42.3)	47 (58.0)	57 (23.0)		

* Comparison of adipose stroma and non-adipose stroma. S, stromal; Bold, *p*-value < 0.05.

**Table 4:** Correlation among the expression of ATX-LPA signaling-related proteins.

Parameters	ATX (S)	LPA1	LPA1 (S)	LPA2	LPA3	LPA3 (S)
ATX						
*r*-value	0.348	0.367	0.125	0.159	−0.036	0.081
*p*-value	**<0.001**	**<0.001**	**0.007**	**0.001**	0.443	0.082
ATX (S)						
*r*-value		0.141	0.352	0.090	−0.023	0.121
*p*-value		**0.002**	**<0.001**	0.051	0.616	**0.009**
LPA1						
*r*-value			0.273	0.221	0.086	0.216
*p*-value			**<0.001**	**<0.001**	0.065	**<0.001**
LPA1 (S)						
*r*-value				0.085	−0.017	0.291
*p*-value				0.066	0.715	**<0.001**
LPA2						
*r* value					0.008	0.079
*p*-value					0.871	0.089
LPA3						
*r*-value						−0.064
*p*-value						0.165

Bold, *p*-value < 0.05.

**Table 5:** Expression of ATX-LPA signaling-related proteins according to CD68- and CD163-positive crown-like structure (CLS) status in adipose stroma.

Parameter	CD68-Positive CLS			CD163-Positive CLS		
	Absent (*n* = 114) (%)	Present (*n* = 23) (%)	*p*-Value	Absent (*n* = 119) (%)	Present (*n* = 18) (%)	*p*-Value
ATX			0.791			0.888
Negative	53 (46.5)	10 (43.5)		55 (46.2)	8 (44.4)	
Positive	61 (53.5)	13 (56.5)		64 (53.8)	10 (55.6)	
ATX (S)			0.063			**0.010**
Negative	101 (88.6)	17 (73.9)		106 (89.1)	12 (66.7)	
Positive	13 (11.4)	6 (26.1)		13 (10.9)	6 (33.3)	
LPA 1			0.910			0.545
Negative	58 (50.9)	12 (52.2)		62 (52.1)	8 (44.4)	
Positive	56 (49.1)	11 (47.8)		57 (47.9)	10 (55.6)	
LPA 1 (S)			**0.017**			0.219
Negative	98 (86.0)	15 (65.2)		100 (84.0)	13 (72.2)	
Positive	16 (14.0)	8 (34.8)		19 (16.0)	5 (27.8)	
LPA 2			0.731			0.665
Negative	70 (61.4)	15 (65.2)		73 (61.3)	12 (66.7)	
Positive	44 (38.6)	8 (34.8)		46 (38.7)	6 (33.3)	
LPA 3			0.222			0.291
Negative	107 (93.9)	23 (100.0)		112 (94.1)	18 (100.0)	
Positive	7 (6.1)	0 (0.0)		7 (5.9)	0 (0.0)	
LPA 3 (S)			**0.004**			**0.009**
Negative	72 (63.2)	7 (30.4)		74 (62.2)	5 (27.8)	
Positive	42 (36.8)	16 (69.6)		45 (37.8)	13 (72.2)	

Bold, *p*-value < 0.05.

**Table 6:** The number of CD68- and CD163-positive macrophages in adipocytes according to the expression of ATX-LPA signaling-related proteins.

Parameter	The Number of CD68-Positive Macrophages in Adipocyte		The Number of CD163-Positive Macrophages in Adipocyte	
	Median (Range)	*p*-Value	Median (Range)	*p*-Value
ATX		0.234		0.526
Negative	5 (0–42)		13 (0–79)	
Positive	1.5 (0–41)		12 (0–62)	
ATX (S)		0.179		**0.003**
Negative	2 (0–42)		12 (0–62)	
Positive	8 (0–32)		20 (0–79)	
LPA1		0.586		0.179
Negative	2 (0–42)		10 (0–79)	
Positive	4 (0–41)		13 (0–62)	
LPA1 (S)		0.066		0.094
Negative	2 (0–42)		12 (0–79)	
Positive	7 (0–37)		14 (0–52)	
LPA 2		0.395		0.943
Negative	3 (0–42)		12 (0–79)	
Positive	3 (0–36)		12.5 (0–57)	
LPA 3		0.350		0.576
Negative	3 (0–42)		12 (0–79)	
Positive	3 (0–9)		12 (0–20)	
LPA 3 (S)		**<0.001**		**<0.001**
Negative	0 (0–41)		8 (0–79)	
Positive	8 (0–42)		16.5 (0–62)	

Bold, *p*-value < 0.05.

**Table 7:** Univariate analysis of the impact of expression of ATX-LPA signaling-related proteins in breast cancers on disease-free survival and overall survival by the log-rank test.

Parameter	Number of Patients/Recurrence/Death	Disease-Free Survival		Overall Survival	
		Mean Survival (95% CI) Months	*p*-Value	Mean Survival (95% CI) Months	*p*-Value
**ATX**			0.842		0.649
Negative	243/13/22	119 (117–122)		117 (114–120)	
Positive	223/13/18	133 (129–136)		130 (125–135)	
ATX (S)			0.093		0.868
Negative	390/25/34	132 (129–134)		129 (126–133)	
Positive	76/1/6	107 (106–108)		104 (101–107)	
LPA1			0.115		0.888
Negative	249/18/22	131 (128–135)		129 (125–134)	
Positive	217/8/18	113 (111–115)		110 (107–112)	
LPA1 (S)			0.152		0.544
Negative	382/24/34	132 (129–135)		129 (125–133)	
Positive	84/2/6	113 (111–115)		111 (108–114)	
LPA 2			0.528		0.413
Negative	409/22/37	133 (131–135)		129 (126–133)	
Positive	57/4/3	110 (105–115)		112 (108–116)	
LPA 3			0.607		0.770
Negative	436/25/38	133 (130–135)		130 (127–133)	
Positive	30/1/2	112 (105–119)		109 (101–118)	
LPA 3 (S)			0.661		0.504
Negative	304/16/28	133 (130–136)		130 (127–133)	
Positive	162/10/12	112 (109–115)		111 (108–114)	

CI, confidence interval.

## Supplementary Materials

Supplementary materials can be found at https://www.mdpi.com/1422-0067/20/9/2102/s1.

## References

[1] Jansen S., Stefan C., Creemers J.W., Waelkens E., Van Eynde A., Stalmans W., Bollen M. (2005). Proteolytic maturation and activation of autotaxin (npp2), a secreted metastasis-enhancing lysophospholipase d. J. Cell Sci..

[2] van Meeteren L.A., Moolenaar W.H. (2007). Regulation and biological activities of the autotaxin-lpa axis. Prog. Lipid Res..

[3] Choi J.W., Herr D.R., Noguchi K., Yung Y.C., Lee C.W., Mutoh T., Lin M.E., Teo S.T., Park K.E., Mosley A.N. (2010). Lpa receptors: Subtypes and biological actions. Annu. Rev. Pharmacol. Toxicol..

[4] Chun J., Hla T., Lynch K.R., Spiegel S., Moolenaar W.H. (2010). International union of basic and clinical pharmacology. Lxxviii. Lysophospholipid receptor nomenclature. Pharmacol. Rev..

[5] Houben A.J., Moolenaar W.H. (2011). Autotaxin and lpa receptor signaling in cancer. Cancer Metastasis Rev..

[6] Willier S., Butt E., Grunewald T.G. (2013). Lysophosphatidic acid (lpa) signalling in cell migration and cancer invasion: A focussed review and analysis of lpa receptor gene expression on the basis of more than 1700 cancer microarrays. Biol. Cell.

[7] Ferry G., Tellier E., Try A., Gres S., Naime I., Simon M.F., Rodriguez M., Boucher J., Tack I., Gesta S. (2003). Autotaxin is released from adipocytes, catalyzes lysophosphatidic acid synthesis, and activates preadipocyte proliferation. Up-regulated expression with adipocyte differentiation and obesity. J. Biol. Chem..

[8] Nikitopoulou I., Oikonomou N., Karouzakis E., Sevastou I., Nikolaidou-Katsaridou N., Zhao Z., Mersinias V., Armaka M., Xu Y., Masu M. (2012). Autotaxin expression from synovial fibroblasts is essential for the pathogenesis of modeled arthritis. J. Exp. Med..

[9] Mao Y., Keller E.T., Garfield D.H., Shen K., Wang J. (2013). Stromal cells in tumor microenvironment and breast cancer. Cancer Metastasis Rev..

[10] Boucharaba A., Serre C.M., Gres S., Saulnier-Blache J.S., Bordet J.C., Guglielmi J., Clezardin P., Peyruchaud O. (2004). Platelet-derived lysophosphatidic acid supports the progression of osteolytic bone metastases in breast cancer. J. Clin. Investig..

[11] Dusaulcy R., Rancoule C., Gres S., Wanecq E., Colom A., Guigne C., van Meeteren L.A., Moolenaar W.H., Valet P., Saulnier-Blache J.S. (2011). Adipose-specific disruption of autotaxin enhances nutritional fattening and reduces plasma lysophosphatidic acid. J. Lipid Res..

[12] Vandeweyer E., Hertens D. (2002). Quantification of glands and fat in breast tissue: An experimental determination. Ann. Anat. Anat. Anz. Off. Organ Anat. Ges..

[13] Ramsay D.T., Kent J.C., Hartmann R.A., Hartmann P.E. (2005). Anatomy of the lactating human breast redefined with ultrasound imaging. J. Anat..

[14] Teo K., Brunton V.G. (2014). The role and therapeutic potential of the autotaxin-lysophosphatidate signalling axis in breast cancer. Biochem. J..

[15] Elston C.W., Ellis I.O. (1991). Pathological prognostic factors in breast cancer. I. The value of histological grade in breast cancer: Experience from a large study with long-term follow-up. Histopathology.

[16] Salgado R., Denkert C., Demaria S., Sirtaine N., Klauschen F., Pruneri G., Wienert S., Van den Eynden G., Baehner F.L., Penault-Llorca F. (2015). The evaluation of tumor-infiltrating lymphocytes (tils) in breast cancer: Recommendations by an international tils working group 2014. Ann. Anat. Anat. Anz. Off. Organ Anat. Ges..

[17] Hammond M.E., Hayes D.F., Dowsett M., Allred D.C., Hagerty K.L., Badve S., Fitzgibbons P.L., Francis G., Goldstein N.S., Hayes M. (2010). American society of clinical oncology/college of american pathologists guideline recommendations for immunohistochemical testing of estrogen and progesterone receptors in breast cancer. J. Clin. Oncol. Off. J. Am. Soc. Clin. Oncol..

[18] Wolff A.C., Hammond M.E., Schwartz J.N., Hagerty K.L., Allred D.C., Cote R.J., Dowsett M., Fitzgibbons P.L., Hanna W.M., Langer A. (2007). American society of clinical oncology/college of american pathologists guideline recommendations for human epidermal growth factor receptor 2 testing in breast cancer. J. Clin. Oncol. Off. J. Am. Soc. Clin. Oncol..

[19] Choi J., Jung W.H., Koo J.S. (2012). Clinicopathologic features of molecular subtypes of triple negative breast cancer based on immunohistochemical markers. Histol. Histopathol..

[20] Goldhirsch A., Wood W.C., Coates A.S., Gelber R.D., Thurlimann B., Senn H.J. (2011). Strategies for subtypes--dealing with the diversity of breast cancer: Highlights of the st. Gallen international expert consensus on the primary therapy of early breast cancer 2011. Ann. Oncol. Off. J. Eur. Soc. Med. Oncol..

[21] Cha Y.J., Kim E.S., Koo J.S. (2018). Tumor-associated macrophages and crown-like structures in adipose tissue in breast cancer. Breast Cancer Res. Treat..

[22] Yang S.Y., Lee J., Park C.G., Kim S., Hong S., Chung H.C., Min S.K., Han J.W., Lee H.W., Lee H.Y. (2002). Expression of autotaxin (npp-2) is closely linked to invasiveness of breast cancer cells. Clin. Exp. Metastasis.

[23] Li T.T., Alemayehu M., Aziziyeh A.I., Pape C., Pampillo M., Postovit L.M., Mills G.B., Babwah A.V., Bhattacharya M. (2009). Beta-arrestin/ral signaling regulates lysophosphatidic acid-mediated migration and invasion of human breast tumor cells. Mol. Cancer Res. MCR.

[24] Popnikolov N.K., Dalwadi B.H., Thomas J.D., Johannes G.J., Imagawa W.T. (2012). Association of autotaxin and lysophosphatidic acid receptor 3 with aggressiveness of human breast carcinoma. Tumour Biol. J. Int. Soc. Oncodev. Biol. Med..

[25] Noh D.Y., Ahn S.J., Lee R.A., Park I.A., Kim J.H., Suh P.G., Ryu S.H., Lee K.H., Han J.S. (2000). Overexpression of phospholipase d1 in human breast cancer tissues. Cancer Lett..

[26] Kitayama J., Shida D., Sako A., Ishikawa M., Hama K., Aoki J., Arai H., Nagawa H. (2004). Over-expression of lysophosphatidic acid receptor-2 in human invasive ductal carcinoma. Breast Cancer Res. BCR.

[27] Bussard K.M., Mutkus L., Stumpf K., Gomez-Manzano C., Marini F.C. (2016). Tumor-associated stromal cells as key contributors to the tumor microenvironment. Breast Cancer Res. BCR.

[28] Choi J., Cha Y.J., Koo J.S. (2018). Adipocyte biology in breast cancer: From silent bystander to active facilitator. Prog. Lipid Res..

[29] Benesch M.G., Tang X., Dewald J., Dong W.F., Mackey J.R., Hemmings D.G., McMullen T.P., Brindley D.N. (2015). Tumor-induced inflammation in mammary adipose tissue stimulates a vicious cycle of autotaxin expression and breast cancer progression. FASEB J. Off. Publ. Fed. Am. Soc. Exp. Biol..

[30] Li M., Xiao D., Zhang J., Qu H., Yang Y., Yan Y., Liu X., Wang J., Liu L., Wang J. (2016). Expression of lpa2 is associated with poor prognosis in human breast cancer and regulates hif-1alpha expression and breast cancer cell growth. Oncol. Rep..

[31] Finak G., Bertos N., Pepin F., Sadekova S., Souleimanova M., Zhao H., Chen H., Omeroglu G., Meterissian S., Omeroglu A. (2008). Stromal gene expression predicts clinical outcome in breast cancer. Nat. Med..

[32] Liu S., Umezu-Goto M., Murph M., Lu Y., Liu W., Zhang F., Yu S., Stephens L.C., Cui X., Murrow G. (2009). Expression of autotaxin and lysophosphatidic acid receptors increases mammary tumorigenesis, invasion, and metastases. Cancer Cell.

[33] Estrella V.C., Eder A.M., Liu S., Pustilnik T.B., Tabassam F.H., Claret F.X., Gallick G.E., Mills G.B., Wiener J.R. (2007). Lysophosphatidic acid induction of urokinase plasminogen activator secretion requires activation of the p38mapk pathway. Int. J. Oncol..

[34] Hao F., Tan M., Xu X., Han J., Miller D.D., Tigyi G., Cui M.Z. (2007). Lysophosphatidic acid induces prostate cancer pc3 cell migration via activation of lpa(1), p42 and p38alpha. Biochim. et Biophys. Acta.

[35] Kalari S., Zhao Y., Spannhake E.W., Berdyshev E.V., Natarajan V. (2009). Role of acylglycerol kinase in lpa-induced il-8 secretion and transactivation of epidermal growth factor-receptor in human bronchial epithelial cells. Am. J. Physiol. Lung Cell. Mol. Physiol..

[36] Lewis C.E., Pollard J.W. (2006). Distinct role of macrophages in different tumor microenvironments. Cancer Res..

[37] Choi J., Gyamfi J., Jang H., Koo J.S. (2018). The role of tumor-associated macrophage in breast cancer biology. Histol. Histopathol..

[38] David M., Ribeiro J., Descotes F., Serre C.M., Barbier M., Murone M., Clezardin P., Peyruchaud O. (2012). Targeting lysophosphatidic acid receptor type 1 with debio 0719 inhibits spontaneous metastasis dissemination of breast cancer cells independently of cell proliferation and angiogenesis. Int. J. Oncol..

[39] Marshall J.C., Collins J.W., Nakayama J., Horak C.E., Liewehr D.J., Steinberg S.M., Albaugh M., Vidal-Vanaclocha F., Palmieri D., Barbier M. (2012). Effect of inhibition of the lysophosphatidic acid receptor 1 on metastasis and metastatic dormancy in breast cancer. J. Natl. Cancer Inst..

[40] Zhang H., Xu X., Gajewiak J., Tsukahara R., Fujiwara Y., Liu J., Fells J.I., Perygin D., Parrill A.L., Tigyi G. (2009). Dual activity lysophosphatidic acid receptor pan-antagonist/autotaxin inhibitor reduces breast cancer cell migration in vitro and causes tumor regression in vivo. Cancer Res..

[41] Benesch M.G., Tang X., Maeda T., Ohhata A., Zhao Y.Y., Kok B.P., Dewald J., Hitt M., Curtis J.M., McMullen T.P. (2014). Inhibition of autotaxin delays breast tumor growth and lung metastasis in mice. FASEB J. Off. Publ. Fed. Am. Soc. Exp. Biol..

