# Supplementary material for: Expression of Autotaxin–Lysophosphatidate Signaling-Related Proteins in Breast Cancer with Adipose Stroma
Source: Int J Mol Sci. 2019 Apr 29;20(9):2102. doi: 10.3390/ijms20092102 (PMC6539826; doi:10.3390/ijms20092102)
Supplement: Supplementary file 1 [file ijms-20-02102-s001.pdf]

Supplementary Figure 1. Expression of LPA2 in tumor cells and LPA3 in stromal cells according to breast cancer stroma type

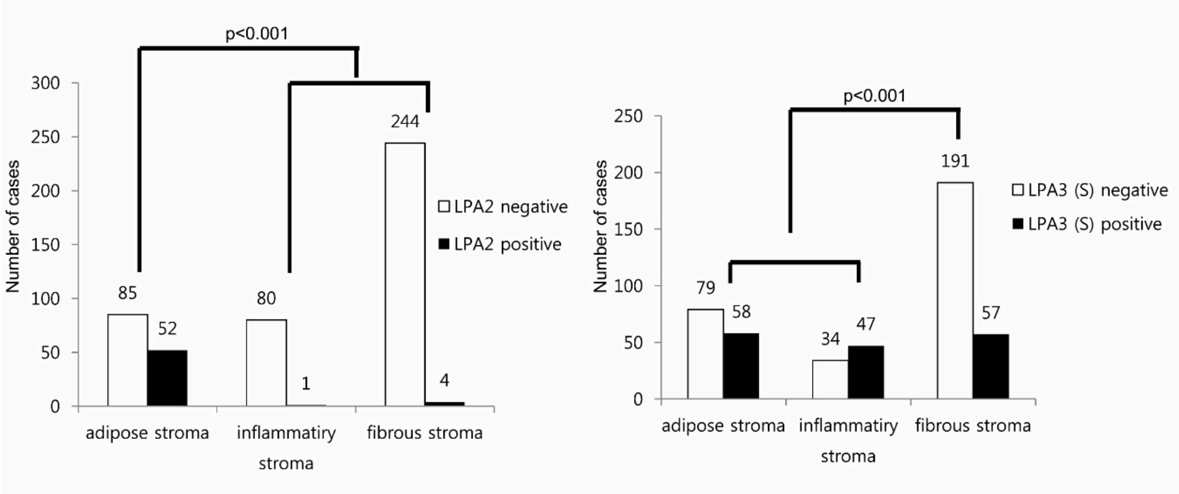

Supplementary Figure 2. Correlation between ATX-LPA signaling-related proteins and clinicopathologic parameters in adipose stroma and non-adipose stroma breast cancer

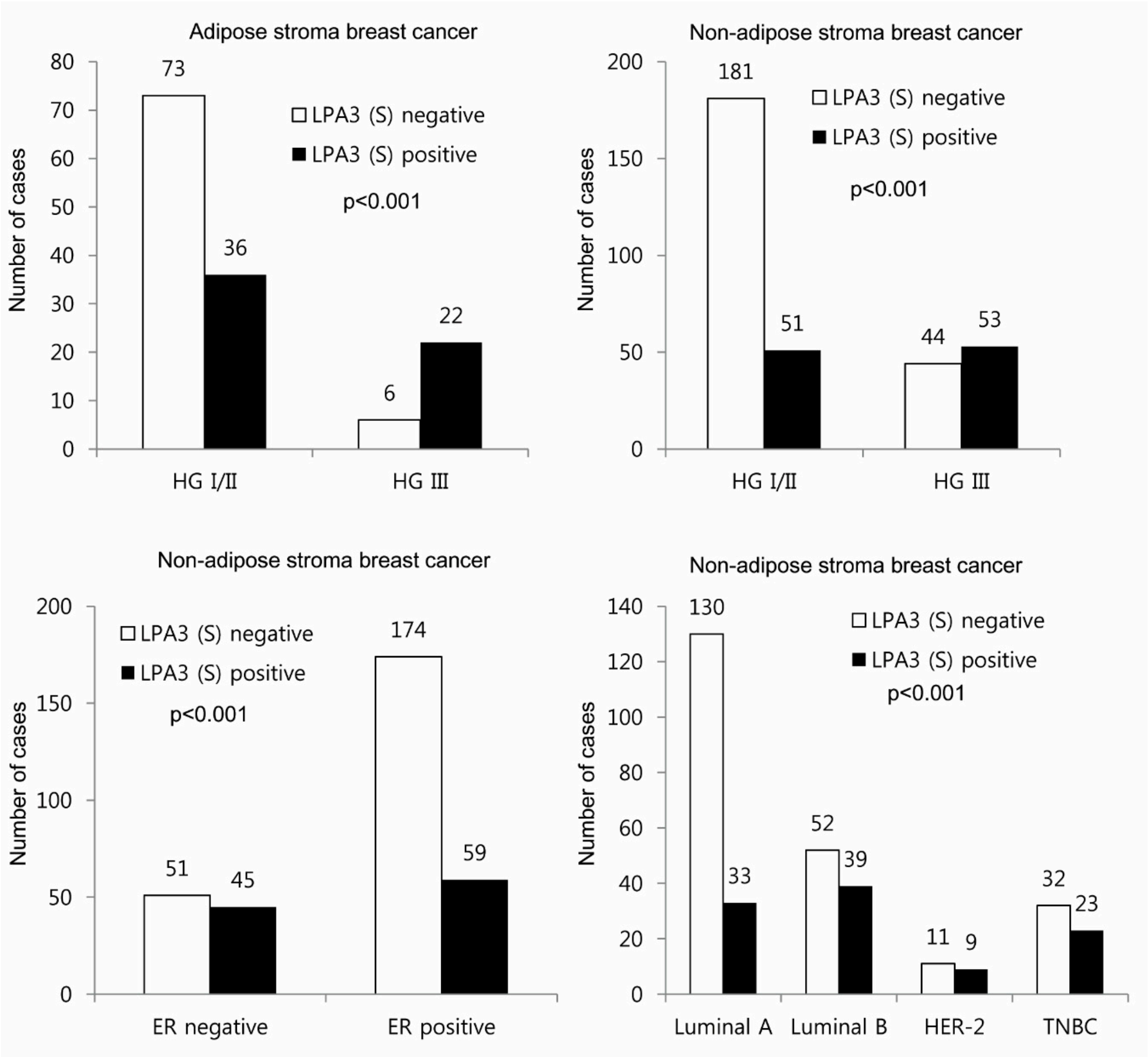
Adipose stroma breast cancer
Number of cases
73
36
6
22
LPA3 (S) negative
LPA3 (S) positive
p<0.001
HG I/II
HG III
Non-adipose stroma breast cancer
Number of cases
181
51
44
53
LPA3 (S) negative
LPA3 (S) positive
p<0.001
HG I/II
HG III
Non-adipose stroma breast cancer
Number of cases
LPA3 (S) negative
LPA3 (S) positive
p<0.001
51
45
174
59
ER negative
ER positive
Non-adipose stroma breast cancer
Number of cases
130
33
52
39
11
9
32
23
LPA3 (S) negative
LPA3 (S) positive
p<0.001
Luminal A
Luminal B
HER-2
TNBC

Supplementary Table 1. Correlation analysis between ATX-LPA signaling-related proteins and clinicopathologic parameters

**Parameter**	**ATX**			**ATX (S)**			**LPA1**			**LPA1 (S)**			**LPA2**			**LPA3**			**LPA3 (S)**		
	**(-)**	**(+)**	***p***	**(-)**	**(+)**	***p***	**(-)**	**(+)**	***p***	**(-)**	**(+)**	***p***	**(-)**	**(+)**	***p***	**(-)**	**(+)**	***p***	**(-)**	**(+)**	***p***
Histologic grade			0.196			0.306			**<0.001**			0.012			0.171			0.194			**<0.001**
I/II	184	157		289	52		200	141		291	50		295	46		316	25		254	87	
III	59	66		101	24		49	76		91	34		114	11		120	5		50	75	
Tumor stage			0.262			0.022			0.617			0.658			0.489			0.351			0.009
T1	128	129		206	51		140	117		209	48		228	29		238	19		181	76	
T2/T3	115	94		184	25		109	100		173	36		181	28		198	11		123	86	
Nodal metastasis			0.216			0.416			0.243			0.563			0.638			0.674			0.291
Absent	140	141		232	49		144	137		228	53		245	36		264	17		178	103	
Present	103	82		158	27		105	80		154	31		164	21		172	13		126	59	
Estrogen receptor			0.130			0.275			0.598			0.141			0.097			0.860			**<0.001**
Negative	67	48		100	15		59	56		89	26		106	9		108	7		57	58	
Positive	176	175		290	61		190	161		293	58		303	48		328	23		247	104	
Progesterone receptor			0.426			0.390			0.668			0.876			0.010			0.198			0.008
Negative	78	64		122	20		78	64		117	25		133	9		136	6		80	62	
Positive	165	159		268	56		171	153		265	59		276	48		300	24		224	100	
HER-2 status			0.300			0.169			0.427			0.126			0.316			0.040			0.013
Negative	206	181		328	59		210	177		322	65		337	50		358	29		262	125	
Positive	37	42		62	17		39	40		60	19		72	7		78	1		42	37	

Molecular subtype			0.589			0.058			0.290			0.057			0.021			0.557			**<0.001**
Luminal A	128	132		228	42		151	119		229	41		223	47		250	20		197	73	
Luminal B	52	53		80	25		50	55		78	27		99	6		99	6		58	47	
HER-2	12	10		19	3		9	13		16	6		20	2		22	0		11	11	
TNBC	41	28		63	6		39	30		59	10		67	2		65	4		38	31	
